# Transient Congenital Hypothyroidism in Turkey: An Analysis on Frequency and Natural Course

**DOI:** 10.4274/jcrpe.2345

**Published:** 2016-06-06

**Authors:** Cengiz Kara, Figen Günindi, Gülay Can Yılmaz, Murat Aydın

**Affiliations:** 1 Ondokuz Mayıs University Faculty of Medicine, Department of Pediatrics, Division of Pediatric Endocrinology, Samsun, Turkey

**Keywords:** congenital hypothyroidism, incidence, iodine deficiency, Neonatal screening, transient hypothyroidism

## Abstract

**Objective:** Prevalence of congenital hypothyroidism (CH) in Turkey at birth was reported to be as high as 1:650 in 2008-2010. Incidence rates of permanent and transient CH separately are unknown due to lack of follow-up data. We aimed to evaluate the impact of transient hypothyroidism on increasing incidence of CH and to determine the natural course and the clinical, biochemical, and imaging characteristics of transient CH.

**Methods:** Baseline and follow-up data of the infants with CH detected at screening in six provinces in the Black Sea Region were analyzed retrospectively during a time period covering the years 2008-2010.

**Results:** Among 138 cases (48% female), 16 (12%) showed transient hyperthyrotropinemia which resolved without intervention. Of the treated 122 cases, 63 (52%) had transient CH. While its frequency was 35% in 2008, it increased to 56% in 2009-2010, following a lowering of the thyroid stimulating hormone cutoff value. The frequency was higher in inland provinces than in coast (67% vs. 43%; p=0.01).Clinical characteristics of permanent and transient cases were similar except female-to-male sex ratios (1.5:1 vs. 0.6:1; p=0.02). L-thyroxine was discontinued in 70% of transient cases before 3 years of age at a median age of 19 (2-36) months. The only indication for early discontinuation of treatment was a low L-thyroxine dose, which was 1.25±0.27 µg/kg/day at withdrawal time.

**Conclusion:** Our regional follow-up data showed that more than half of newborns with primary CH had transient thyroid dysfunction. In the majority of cases, discrimination between transient and permanent CH can be made before age 3 years, as indicated by cessation of L-thyroxine treatment.

## WHAT IS ALREADY KNOWN ON THIS TOPIC?

Birth prevalence of primary congenital hypothyroidism (CH) was found to be very high in Turkey after the national screening. However, the incidence rates of permanent and transient CH are unknown due to lack of follow-up data. To distinguish between transient and permanent forms, the guidelines recommend that the children with unexplained CH be re-evaluated after 3 years of age through a trial of treatment withdrawal.

## WHAT THIS STUDY ADDS?

Transient hypothyroidism exists in more than half of the newborns detected at screening in our region and most probably throughout the country. Lowering of thyroid stimulating hormone cutoffs has led to increased birth prevalence owing to detection of a higher number of mild cases of both permanent and transient CH. In the majority of cases, discrimination between transient and permanent CH could be possible before 3 years of age. This study has shown that unnecessary treatment of transient CH can be avoided in many infants owing to early cessation of low dose L-thyroxine.

## INTRODUCTION

Congenital hypothyroidism (CH) is one of the most common causes of mental retardation that can be prevented by early detection and treatment. Many developed countries have largely eliminated intellectual disability caused by CH owing to newborn screening programs (NSPs) (1). In Turkey, the National Newborn Screening Program (NNSP) for CH was started at the end of 2006 by the Turkish Directorate of Public Health. In 2013, Dilli et al (2) published the first NNSP data that showed very high CH incidence rates (1:888 in 2008, 1:592 in 2009, and 1:469 in 2010). The overall incidence rate of CH during this period (2008-2010) was 1:650.

The data cited above reflect the birth prevalence of primary CH, which might be potentially permanent or transient. Several studies (3,4,5,6,7) showed that the frequency of transient CH in Turkey ranged between 25-65%, indicating that transient cases might have played a substantial role in the high incidence of CH. Unfortunately, a nationwide registry that collects information on the results of re-evaluation of CH diagnosis at the follow-up centers is not yet available in Turkey. For that reason, current incidence rates for permanent and transient CH are unknown. A recent study reported for the first time that among cases detected at national screening, the frequency of transient CH was 30% (7). However, this study was not intended to be representative of a region. Therefore, there is still no nationwide or regional follow-up data that can be used to estimate the incidence rates for permanent and transient CH.

Transient primary hypothyroidism is a heterogeneous disorder that may be caused by iodine deficiency or excess, maternal thyroid stimulating hormone (TSH) receptor blocking antibodies, maternal antithyroid drugs, and genetic defects such as dual oxidase 2 (DUOX2) and TSH receptor mutations (8,9). However, the underlying mechanism remains often unknown (10,11). To discriminate permanent and transient forms, the guidelines recommend that children with unexplained CH with gland in situ be re-evaluated after 3 years of age through a trial of treatment withdrawal (12,13). However, some cases of transient CH caused by identifiable factors such as iodine deficiency or excess may also frequently require a short-term therapy. The prevalence of transient CH in Turkey is much higher than the expected rate of 5-10% reported for iodine-sufficient populations (14). Numerous studies carried out in newborns (15,16,17) and pregnant women (18,19,20) have already demonstrated that iodine deficiency is a continuing problem of Turkey. Also, a recent study revealed that iodine excess could be a problem in newborns due to use of iodine-containing antiseptics during delivery despite maternal iodine deficiency (20). Overall, little is known about the natural course of transient CH in Turkey (6,7). It is possible that treatment withdrawal before 3 years of age may be more commonly applicable.

The aims of our study were to evaluate the impact of transient hypothyroidism on the increasing incidence of CH as well as to investigate the natural course and clinical, biochemical, and imaging characteristics of transient CH in the cases detected at national screening in Turkey.

## METHODS

**Study Area:** The Turkish Statistical Institute (TSI) publishes demographic data by dividing Turkey into 12 regions (http://www.tuik.gov.tr/). Our study area includes six neighboring provinces comprising Samsun, Amasya, Tokat, Çorum, Sinop, and Ordu. With the exception of Ordu which is a province on the eastern part of the Black Sea coast, these provinces are located in the West Black Sea Region, which is one of the 12 regions of Turkey. Of the whole population in the West Black Sea Region and Ordu, 70% (n=3 645 836) live in our study area. The Clinic of Pediatric Endocrinology at Ondokuz Mayıs University in Samsun is a tertiary referral center for the patients residing in this area.

**Potential CH Cases in the Area:** According to the NNSP data (2), the birth prevalence of CH in the West Black Sea Region was about 1:600 between 2008 and 2010. Also, according to the annual birth statistics of six provinces obtained from the website of TSI, the total number of live-born infants was 157 839 during the years of 2008-2010. By multiplying the birth prevalence of CH (1:600) and the number of live-born infants (157 839), we have estimated that around 263 CH cases should have been detected at screening in our area during the study period. While three provinces (Samsun, Ordu, Sinop) in our region are on the seaside, the other three (Amasya, Tokat, Çorum) are with inland location. According to TSI data, 59% of the infants were born in the coastal provinces and the remaining 41% were born in the inland area. Therefore, the potential CH cases should have been scattered proportionally between coastal and inland provinces (Table 1).

**Figure 1:**
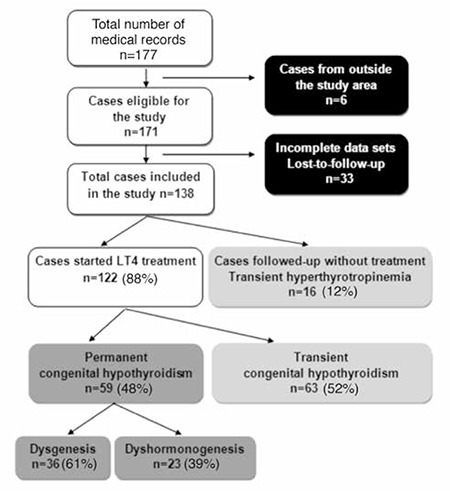
Flow diagram of the follow-up study

**Patients:** After approval of the Ondokuz Mayıs University Institutional Ethics Committee, we carried out a retrospective analysis of the medical records of patients who were consecutively diagnosed as CH in our clinic between January 2008 and December 2010. These patients were transferred to our clinic by the Provincial Directorates of Public Health carrying out the NNSP. Patients born and resident in the 6 provinces listed above were included in the study. Patients who were from outside the study area (n=6), those who had incomplete data sets (n=12), and those who were lost to follow-up before discrimination between transient and permanent CH (n=21) were excluded from the study. Therefore, the study cohort consisted of 138 cases with CH (Figure 1).

**Study Protocol:** The newborns with a high serum TSH level (>10 µU/mL) were referred to our institution by the NNSP team as suspected primary CH cases (2). Based on the serum levels of TSH and free thyroxine (fT4) measured at our endocrine clinic (or at a local hospital for a few infants who had been referred after initiation of treatment), the patients were classified as primary overt (TSH>10 µU/mL, fT4<0.9 ng/dL) or subclinical CH (TSH>10 µU/mL, fT4≥0.9 ng/dL) (21). L-thyroxine (L-T4) treatment (in a dose of 8-15 μg/kg) was started in patients with low fT4 and/or high TSH levels (fT4<0.9 ng/dL and/or TSH>25 µU/mL) (21). Infants with normal fT4 and slightly elevated TSH (10-25 µU/mL) were followed-up without treatment and rechecked at 2-week intervals. If serum TSH concentration was found to be >25 µU/mL at any time or >10 µU/mL after 4-6 weeks of age, these infants were also started on L-T4 treatment (12,21). On the other hand, infants whose TSH levels returned to normal within 4-6 weeks without intervention were considered to have transient hyperthyrotropinemia (Figure 1) (12,21).

At baseline or during follow-up, CH was categorized as permanent or transient. The criteria for a diagnosis of permanent CH were: 1- absent or ectopic thyroid on imaging; or 2- an increase in medication dosage over time or elevated TSH due to non-compliance to treatment; or 3- elevated TSH (>10 µU/mL) after a 30-day trial off L-T4 therapy at age ≥3 years (12,22,23). If a suppressed serum TSH level was observed despite low doses of L-T4, a trial off medication was done before 3 years of age in the cases with ectopic glands. The presence of normal thyroid function tests over 6 months following a withdrawal of L-T4 was considered to indicate transient CH (22).

In order to determine the distinguishing characteristics and natural course of transient hypothyroidism, the infants with permanent and transient CH were evaluated comparatively in terms of demographic, clinical, laboratory, and treatment parameters at baseline and during follow-up. In addition, to assess the impact of TSH cutoff and geography on the frequency of transient CH, the cases were also evaluated by year (2008 vs. 2009-2010) and by province (inland vs. coastal).

**Laboratory and imaging methods:** Serum TSH, fT4, and thyroglobulin levels were measured by electrochemiluminescence immunoassay using Elecsys 2010 modular analytics E170 (Roche Diagnostics, Indianapolis, IN, USA). Reference ranges of TSH, fT4, and thyroglobulin for infants at ages between 6 days and 3 months were 0.72-11.0 µU/mL, 0.9-2.2 ng/dL, and 20-228 ng/mL, respectively. Thyroid ultrasound (US) was performed with an Aplio XG SSA-790A US scanner (Toshiba Medical Systems Co., Tokyo, Japan) and a 12-MHz linear-array transducer. The volume of thyroid lobes was calculated using the following formula: V (mL)=0.479xlengthxwidthxthickness (all in cm). Total thyroid volume was the sum of the volumes of two lobes (24). Thyroid gland was considered goitrous when its total volume exceeded +2 standard deviation (SD) score of the volume found by Kurtoglu et al (25) for Turkish newborns of different gestational ages. Thyroid scintigraphy (scan) with 99mTc-pertechnetate was performed using a Siemens Orbiter gamma camera equipped with a pinhole collimator (Siemens, Erlangen, Germany).

**Statistical Analysis**

The data were analyzed using SPSS software (Statistical Package for the Social Sciences, version 15.0, SPSS Inc, Chicago, IL, USA). Values in the text were presented either as means ± SD or, if not normally distributed, as medians (ranges). Statistical analysis was performed using parametric (chi-square and student’s t-test) or non-parametric (Fisher exact and Mann-Whitney U) tests, when appropriate. A p-value <0.05 was accepted as statistically significant.

## RESULTS

Table 1 shows distribution of the live-born infants, the potential CH cases, and the patients with permanent and transient CH in our study group by geography, namely, by provinces located at seaside and inland. According to the numbers of live-born infants, 59% of the potential CH cases should have been detected in the coastal provinces. Of the patients in our study group, 63% came from the coastal area. A comparison between expected and observed ratios of CH infants by the provinces did not show a significant difference (p=0.48), indicating that our study group was representative for all potential CH cases living in both seaside and inland. Also, there was no significant difference among the frequencies of transient CH in the six provinces, ranging between 40% and 73% (p=0.19). However, the frequency of transient CH was higher in the inland provinces compared to that in the coastal ones (67 vs. 43%, p=0.01).

Figure 1 shows a flow diagram of the follow-up study. After exclusion of 33 cases due to incomplete data set and lack of follow-up, a total of 138 children were included in the study. Demographic characteristics (birthplaces, sex ratios, and median ages at presentation) of the excluded infants were not different from those of the study cases (data not shown). Thus, among 263 probable cases of CH detected in our area, 65% (n=171) were evaluated in our clinic, and 53% (n=138) were included in the study. As depicted in Figure 1, sixteen (12%) of these cases showed transient hyperthyrotropinemia. Among 122 cases treated with L-T4, 63 (52%) were found to have transient CH. Permanent CH was identified in 48% of the cases.

All treated infants underwent a thyroid scan and/or an US (53 scan, 46 US, and 21 both) at presentation (n=119) or after 3 years of age (n=3). Based on the findings of thyroid imaging, the etiology of permanent CH was classified as follows: dysgenesis, 61% (22 ectopia, 10 agenesis, 4 hypoplasia) and dyshormonogenesis, 39% (14 normal and 9 hyperplastic glands). In transient CH cases, thyroid imaging revealed a normal (n=34), hyperplastic (n=26), or hypoplastic (n=3) gland in situ. Three patients including one transient case showed a discordant result; while a thyroid gland was reported to be present at US, the scan revealed no uptake (Table 2). Maternal autoimmune thyroiditis was reported in 6 cases (3 transient and 3 permanent). Two of them were receiving L-T4 therapy, whereas there was no history of antithyroid or iodine-containing drug use.

The baseline clinical characteristics of the cases with permanent and transient CH are shown in Table 2. Our cohort consisted of 59 females and 63 males, giving a female-to-male sex ratio of 0.9:1. While the female-to-male ratio was 1.5:1 in the permanent group, this ratio was 0.6:1 in the transient group (p=0.02). The female-to-male ratio was higher in the permanent CH cases with thyroid dysgenesis (1.8:1) than those with dyshormonogenesis (1.1:1). There were no statistically significant differences between permanent and transient CH groups in terms of birth weight, gestational age, frequency of prematurity, and median ages at presentation and starting therapy.

The mean serum TSH level was higher in the permanent group than the transient one (91±62 vs. 67±33 µU/mL, p=0.009). But, the dispersion of TSH levels largely overlapped in both groups [100 (12-420) vs. 69 (10-100) µU/mL]. Mean fT4 levels were equally low in the two groups. Also, the frequencies of overt and subclinical hypothyroidism were not different between the two groups. However, serum thyroglobulin levels were higher in the cases with transient CH compared to those with permanent CH (241±90 vs. 134±113 ng/mL, p=0.001). Within the permanent CH group, serum thyroglobulin levels of the patients with dysgenesis and dyshormonogenesis were 80±89 and 192±112 ng/mL, respectively (p=0.01). A comparison between the patients with transient CH and permanent dyshormonogenesis did not show a significant difference (p=0.48).

The whole blood TSH cutoff value was 10 µU/mL in 2008 and was lowered to 7.5 µU/mL in 2009 (2). In our study group, the number of permanent and transient CH cases has gradually increased from 2008 to 2010, but the increment in the transient group has been more evident (Table 3). The frequency of transient CH was significantly higher in the years of 2009-2010 than in the previous year at which time TSH cutoff was higher (56 vs. 35%, p=0.05).

Table 4 summarizes the follow-up and treatment data of the cases with permanent and transient CH. L-T4 therapy was discontinued at a median age of 19 months (ranges 2-36 months) in the cases with transient CH. While 54% of the infants with permanent CH were diagnosed at presentation owing to abnormal thyroid imaging, 27% received a final diagnosis during follow-up due to either increased L-T4 dosage over time or elevated TSH caused by non-compliance to treatment. Only 19% of permanent cases required a trial off medication after 3 years of age for definitive diagnosis. On the other hand, L-T4 was discontinued before 3 years of age in 70% (n=44) of transient cases. Finally, L-T4 doses were significantly lower in the transient CH cases than the permanent ones at the onset of treatment (p=0.03) and at the 6 months and thereafter (p<0.001). In the transient CH cases (n=44) whose treatment was discontinued early, L-T4 dose was 1.25±0.27 µg/kg/day at the time of withdrawal. All of them were receiving L-T4 at a dose of <25 µg/day (39 patients, 12.5 µg/day; 2 patients, 18.75 µg/day, and 3 patients 6.25 µg/day).

## DISCUSSION

The results of our study have revealed that 52% of the cases detected at screening in our area had transient CH apart from transient hyperthyrotropinemia resolved without intervention. Also, the present study showed that in 70% of transient CH cases, L-T4 treatment was discontinued before 3 years of age, at a median age of 19 months.

Birth prevalence of primary CH was reported as 1:650 in Turkey during the period of 2008-2010 (2). This prevalence was around 1:600 in the West Black Sea Region comprising our study area. However, due to lack of follow-up data, it is still unknown how transient hypothyroidism has made a contribution to the high incidence rate of CH. Owing to a coverage ratio of 65%, we believe that our study data can be used to estimate the incidence rates of permanent and transient CH in our region. If transient CH cases of 52% are excluded, the birth prevalence that is 1:600 will be reduced to nearly 1:1250 that corresponds to the incidence of permanent CH in our region. Through a similar approach, the incidence of transient CH can be estimated as 1:1154.

Prior to the onset of NNSP, the regional studies from Turkey reported the incidence of CH in a range of 1:2736 to 1:2326 (3,26,27). The NNSP data indicated that the incidence of CH has increased dramatically over the last two decades. In fact, several NSPs around the world have reported approximately a twofold increase in the incidence of CH (1). Lowering of the screening TSH cutoffs in these programs has been associated with the doubling of CH incidence, primarily explained by detection of milder cases. While the whole blood TSH cutoff value in Turkey was 20 µU/mL in the past (3,26,27), it was chosen as 10 µU/mL at the start of NNSP, and was lowered to 7.5 µU/mL in 2009 (2). With the reduction of TSH cutoff levels, an increase in CH incidence would be expected, but in Turkey the increase has been beyond that expected (approximately 4-fold) compared to other countries including Italy, England, and Greece (28,29,30). Thus, the rise in CH incidence in Turkey was not explicable in the basis of an enhanced detection of milder cases of true CH alone. Our study has shown that transient and permanent forms of hypothyroidism have contributed jointly to the increased CH incidence in our region. Comparison of the previous 1:2326 (3) and the current estimated incidence rates (1:1250) indicates a nearly 2-fold increase in permanent CH incidence in the West Black Sea Region. This is compatible with a worldwide trend towards doubling of CH incidence, which is usually explained by the lower TSH cutoff level (1,28,29,30).

On the other hand, the impact of lowering of the TSH cutoff on the incidence of transient CH has been more dramatic with an approximate 5-fold increase, which is calculated by comparing the earlier incidence (1:6202 in Ref. 3) and our current estimation (1:1154). The frequency of transient CH was 27% in our region in the period of 2000-2002 (4). Our study revealed that this frequency has increased to 35% in 2008 and to 56% in 2009-2010. Such a high increment in the frequency and incidence of transient CH has not been observed in other European countries nor in the USA (28,30,31). However, in Iran, where the cutoff level for TSH is 5 µU/mL, the birth prevalence of CH was recently reported as high as 1:307 (32). After re-evaluation, the incidence rates of permanent and transient CH were 1:581 and 1:628, respectively. The frequency of transient CH in Markazi Province of Iran was 48%, a ratio similar to that (52%) in our cohort. In Turkey, Dilli et al (2) reported the birth prevalence of CH as 1:469 for the year 2010. Because the TSH cutoff was recently lowered to 5.5 µU/mL whole blood in NNSP, it will not be surprising to see a report on a much higher birth prevalence of CH in the near future in Turkey, similar to Iran. In short, the rate of transient CH in our country is comparable to that reported for Iran, our neighbor in the Middle East, but it is much higher than the rates reported for European countries and the USA.

Past experience in Italy showed that the frequency of transient hypothyroidism was 58% in the high CH incidence (>1:2000) areas affected historically by iodine deficiency. As CH incidence decreased, lower percentages of transient CH were observed in several districts of Italy (33). In Turkey, the 2010 NNSP data revealed very high CH incidence rates varying between 1:996 and 1:250 in 12 regions (2). In our study region that was shown to be affected by mild-to-moderate iodine deficiency in the past (3,34), CH incidence was 1:418 in 2010 (2), and we found that 57% of this could be attributed to transient cases. In accordance with the previous Italian experience, these data suggest a continuing iodine deficiency problem in Turkey.

In fact, transient CH in Turkey is an old problem and its frequency varies between 25-65% from one region to another (3,4,5,6,7). The present study has shown that the frequency of transient CH ranged between 40% and 75% in six provinces in our area, indicating that it was largely affected by the geography. While the frequency was 40% to 45% among infants from the coastal provinces, it was as high as 60% to 75% among those in the inland provinces. Given that the frequency of transient CH is between 5-10% in iodine-sufficient populations (14), the ratios varying between 25-75% reported in the present and other studies possibly reflect variations in iodine status in different regions of Turkey.

Before salt iodization program implemented was initiated in 1998, Turkey was a mild to severe iodine deficiency area (34,35). After iodization program, based on the results of monitoring studies on urinary iodine concentration among school-age children, iodine deficiency has been considered to be eliminated in most urban areas of Turkey (34,36). The World Health Organization has previously recommended the measurement of neonatal TSH in addition to urinary iodine as an indicator for population iodine status. The criterion for iodine sufficiency is that the frequency of whole blood TSH values >5 µU/mL should be under 3% in a population (37). The NNSP data of 2010 showed that the recall rate for the whole blood TSH cutoff of 7.5 µU/mL is 3.8% (2). It is obvious that the rate of newborns with a screening TSH value >5 µU/mL would be much greater than 3% in Turkey. Some experts have proposed that the data on neonatal TSH screening for monitoring population iodine deficiency should be interpreted with caution due to technical issues including the time of sampling after birth (38). Nevertheless, many studies have shown an inverse relationship between neonatal TSH and maternal urinary iodine concentration, supporting the notion that the frequency of neonatal TSH concentrations >5 µU/mL was a sensitive indicator of iodine nutrition during pregnancy (39,40,41). In recent years, several studies have already demonstrated insufficient iodine intake of pregnant women living in different regions of Turkey (17,18), including apparently iodine-sufficient areas (19). As a result, the finding of the high frequency of transient CH is in line with the high recall rate in the NNSP. These data should be interpreted also considering nutritional iodine deficiency in pregnant women, which is clearly different from the iodine status among school-age children in Turkey.

Beside iodine deficiency, other environmental and genetic factors might have contributed to the occurrence of transient CH in our cohort. Iodine excess induces transient hypothyroidism by the Wolff-Chaikoff effect lasting usually about 10 days, and it can be caused by the use of iodine-containing antiseptics, contrast agents and amiodarone (8). In our study group, there was no history of exposure to antithyroid or iodine-containing drugs. However, topical iodine exposure cannot be excluded as a cause of transient CH in our cohort. In fact, newborns in iodine-deficient regions might be more susceptible to the Wolff-Chaikoff effect of topical iodine exposure (9). In a recent study carried out in Zonguldak, a city in our region, iodine excess was observed in 61% of 116 healthy newborns, while their nursing mothers showed a iodine-deficient nutritional status (20). In that study, the recall rate at screening was found to be 9.5% and three newborns required L-T4 therapy. Iodine excess in newborns was attributed to the use of iodine-containing antiseptics during delivery. This same study has demonstrated that iodine deficiency remained an unresolved problem in nursing mothers in our region, and that iodine excess contributed to the high recall rates, as well the increased incidence of transient CH and hyperthyrotropinemia (20).

Maternal TSH receptor blocking antibodies may lead to transient hypothyroidism, but this is a rare condition that occurs in 1-2% of all newborns with CH or 1 in 180,000 live births (42). Under conditions of exposure to maternal blocking antibodies or excess iodine, the thyroid usually can be identified in a normal location by ultrasonography, but radioisotope uptake might be blocked partially or completely (9,14). In our study, only one case in the transient group has shown such discordance between US and scan, implying that blocking antibodies or excess iodine are not major contributing factors. The thyroid gland was enlarged in 41% of the newborns with transient CH. In addition, as compared to the patients with permanent CH, transient CH cases had higher serum thyroglobulin levels despite the lower TSH and the equally low fT4 levels. This biochemical profile together with a significant proportion of goiter supports a possibility of iodine deficiency in our cohort. However, high thyroglobulin levels and goiter may be also caused by iodine organification defects, which have been detected in nearly 20% of the patients with transient CH (10). The etiology remains unexplained in the majority of such patients albeit DUOX2 mutations were demonstrated in some (10).

Mutations in thyroid peroxidase (TPO), DUOX2 and TSH receptor genes may cause permanent or transient CH (8,9,10,43). In the present study, thyroid imaging showed normal, enlarged, or hypoplastic gland in situ in 46% of the patients with permanent CH, indicating a higher possibility of recessively inherited genetic defects in our region where consanguineous marriages were relatively frequent. Therefore, genetic background might also have contributed to the development of transient thyroid dysfunction in our cohort. Given that iodine intake may alter the phenotype of TPO and DUOX2 mutations causing iodine organification defects (8,43,44,45), it is even possible that iodine deficiency in our region might have increased the expressivity of gene defects. In Turkey, permanent CH due to dyshormonogenesis is mainly caused by TPO mutations (46), but there is no study investigating the genetic background in transient CH.

As another important finding, the present observational study has shown that early discrimination between transient and permanent CH could be possible in the majority of children with gland in situ. This finding was consistent with the data of latest reports (11,47,48). Imaging studies revealed ectopic gland or athyreosis in only 25% of the cases with CH, and thereby let us to make a definitive diagnosis on admission in a small group. The majority of patients who had an ectopic gland received a final diagnosis during follow-up. In transient group, no case required an increment in L-T4 doses and therapy was discontinued at a median age of 19 months with a range of 2-36 months. Thus, we reached a definitive diagnosis in 70% of transient cases before 3 years of age, the recommended time for re-evaluation of the thyroid axis through a trial off treatment (12,13). For discrimination between transient and permanent CH patients with gland in situ, there was no clinical or laboratory parameter including gestational age, birth weight, presentation time, age at onset of therapy, or the degree of hypothyroidism based on serum levels of TSH, fT4, and thyroglobulin. The only indication for early discontinuation of treatment was the L-T4 dose. We could stop therapy early owing to low doses of L-T4 (usually <1.5 µg/kg/day or <18.75 µg/day). This experience is in agreement with the data of a recent study from Turkey, which has shown that L-T4 dose is the sole criterion that can be used to distinguish permanent and transient CH (49). Messina et al (48) proposed that L-T4 requirements <1.7 µg/kg/day at 12 months or <1.45 µg/kg/day at 24 months were highly suggestive of transient CH. On the other hand, Cho et al (47) suggested that infants with CH requiring L-T4 doses <3.25 µg/kg/day at 12 and 24 months were likely to have transient CH. In our study, mean L-T4 doses among the transient CH cases were 2.0±0.7 and 1.6±0.3 µg/kg/day at 12 and 24 months, respectively. In the patients whose treatment was stopped before age 3 years, the dose was 1.25±0.27 µg/kg/day at the time of withdrawal. Although the mean duration of treatment in our cohort was shorter than the recommended usual time, we believe that L-T4 substitution could possibly have been discontinued even earlier, at least in some patients.

In cases with transient hypothyroidism caused by easily identifiable factors including maternal thyroid diseases and iodine deficiency or excess, L-T4 treatment is frequently stopped within the first year of life (11,14). Although iodine intake appears to be the most likely explanation for the high frequency of transient CH in our region, 75% of transient cases were still receiving L-T4 treatment at the end of the first year. Hence, other mechanisms, especially monogenic defects might have played a role in the development of transient CH. Nevertheless, it must be pointed out that even in iodine-sufficient populations, the underlying mechanism of transient CH remains unexplained in the majority of the cases (10,11,22,23). The major limitation of our study was the lack of direct evidence on possible explanations for transient CH including iodine status and genetic background of the patients. This limitation was a result of the retrospective design of the study. Therefore, additional prospective studies will be necessary to investigate iodine status and known or possibly novel genetic defects in the CH population in Turkey. But during this period, to reduce the high frequency of transient CH, it appears reasonable to suggest that pregnant women be supplemented with iodine-containing preparations in addition to iodized salt consumption in iodine-deficient areas in Turkey.

In conclusion, our study showed that more than half of the newborns with primary CH had transient thyroid dysfunction. Lowering of TSH cutoffs has led to the increased birth prevalence of CH owing to the detection of a higher number of mild cases of both permanent and transient CH. In the majority of cases, discrimination between transient and permanent CH appears to be possible before age 3 years.

**Ethics**

Ethics Committee Approval: Ondokuz Mayıs University Institutional Ethics Committee 2012, Informed Consent: It was taken.

Peer-review: External peer-reviewed.

## AUTHORSHIP CONTRIBUTIONS

Concept: Cengiz Kara, Murat Aydın, Design: Cengiz Kara, Murat Aydın, Data Collection and/or Processing: Figen Günindi, Gülay Can Yılmaz, Analysis and/or Interpretation: Cengiz Kara, Figen Günindi, Literature Research: Cengiz Kara, Figen Günindi, Writing: Cengiz Kara.

Financial Disclosure: The authors declared that this study has received no financial support.

## Figures and Tables

**Table 1:**
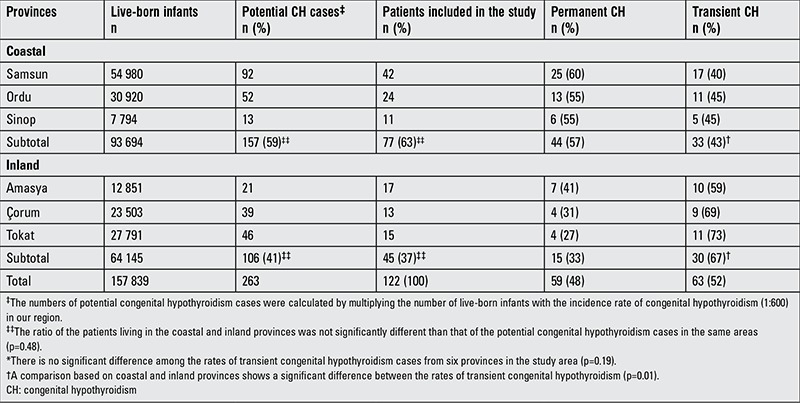
Distribution of the live-born infants, potential congenital hypothyroidism cases, and patients with permanent and transient congenital hypothyroidism by provinces (located at seaside and inland)*

**Table 2:**
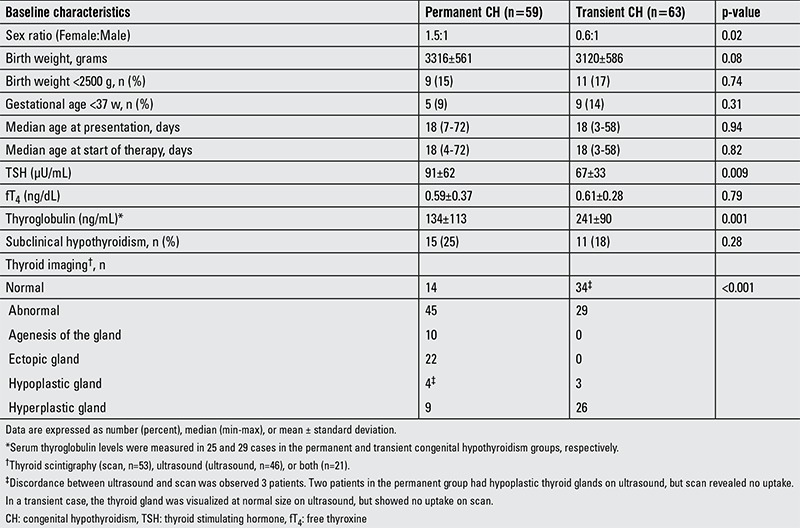
Baseline characteristics of infants with permanent and transient congenital hypothyroidism

**Table 3:**
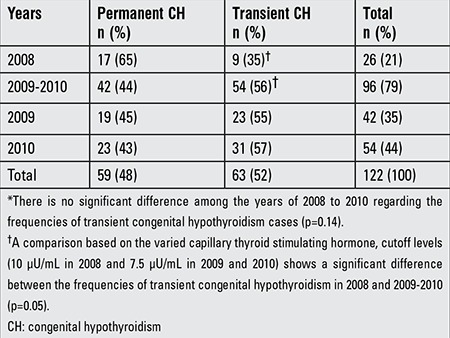
Distribution of the permanent and transient congenital hypothyroidism cases by years*

**Table 4:**
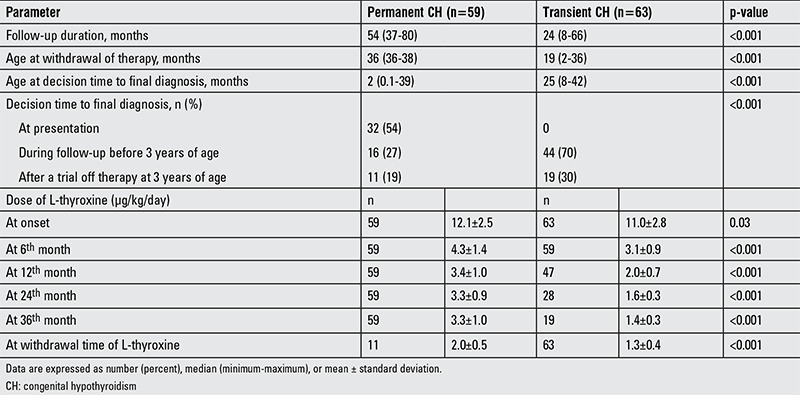
Follow-up and treatment data of the cases with permanent and transient congenital hypothyroidism
